# Lymphoplasmacyte-rich meningioma in the central nervous system: An unusual case report

**DOI:** 10.1097/MD.0000000000027991

**Published:** 2021-12-30

**Authors:** Han Wang, Bin He, Yuelong Wang, Haifeng Chen, Siqing Huang, Jianguo Xu

**Affiliations:** a Department of Neurosurgery, West China Hospital, West China Medical School, Sichuan University, Chengdu, PR China; b Department of Obstetrics, West China Second Hospital, Sichuan University, Chengdu, PR China; c Department of Neurosurgery, the second People's Hospital of Yibin, Yibin, Sichuan Province, China.

**Keywords:** case report, immunotherapy, lymphoplasmacyte, meningioma, surgical resection

## Abstract

**Rationale:**

Lymphoplasmacyte-rich meningioma (LPRM) is a rare meningioma characterized by significant infiltration of plasma cells and lymphocytes, and changes in the ratio of meningeal epithelial components. According to the World Health Organization, tumors of the central nervous system are classified as grade I tumors.

**Patient concerns:**

A 44-year-old man presented to our department with complaints of limb weakness accompanied by hand numbness. Half a month before admission, the patient's limb weakness worsened and he could not walk and raise his hands, with limb sensory disturbance and incontinence.

**Diagnosis:**

Magnetic resonance imaging of the head and cervical spinal cord showed a diffuse extramedullary mass creeping on the tentorium and skull base meninges along the clivus down to the sixth cervical spinal meninges. The cervical spinal cord was enveloped and pressed (Fig. 1A-C). Postoperative histopathological examination showed meningothelial areas admixed with lymphocytes and plasma cells (Fig. 2D-H), indicating that the mass was a LPRM.

**Intervention:**

Suboccipital craniotomy, C1 laminectomy, and C2-C6 laminoplasty were performed for this patient, and postsurgical pathology showed that the tumor was a LPRM with large amounts of lymphocytes and plasma cells.

**Outcome:**

After 2 weeks of active treatment, the patient died of worsening pneumonia.

**Lessons:**

LPRM is a rare variant of meningioma, and it is more unusual that the lesions involve the intracranial dura mater and the entire cervical spinal meninges. So far, surgical resection has been the main treatment for LPRM, but according to its own characteristics of lymphoplasmacyte-rich, immunotherapy may become a new treatment option.

## 1 Introduction

Meningiomas are the second most common primary brain tumors in adults, accounting for more than 30% of primary brain tumors.[^1^,^2^] According to the World Health Organization (WHO) (2016) classification of tumors of the central nervous system (CNS), meningiomas are divided into 15 subtypes. Lymphoplasmacyte-rich meningioma (LPRM) is an uncommon pathological subtypes.^[3]^ Meningiomas usually attach to the inner surface of the dura mater, and the most common locations are the dural reflex, such as the cerebral falx, tentorium cerebelli, and venous sinuses.^[4]^ However, it is rare that the lesions invade the spinal dura mater; to the best of our knowledge, this is the first reported case of lymphoplasmacyte-rich meningioma with the broadest range, which involves the saddle area, tentorium cerebella, and along the meninges of the skull base from the clivus down to the sixth cervical spinal cord.

## 2 Case presentation

### 2.1 Clinical presentation

A 44-year-old man presented to our department with complaints of limb weakness accompanied by hand numbness. Half a month before admission, the patient's limb weakness worsened and he could not walk and raise his hands, with limb sensory disturbance and incontinence; there was a history of appendectomy 20 years ago, oblique inguinal hernia repair 5 years ago, and bilateral hearing weakness for 3 years. When the patient was admitted to our department, a catheter was placed. Motor and sensory examinations revealed upper limb paralysis of grade 2/5 and lower limb paralysis of grade 4/5 upper motor type, and hypoesthesia below the C2 level. The bilateral Babinski signs were positive.

### 2.2 Diagnosis and preoperative course

**Figure 1:**
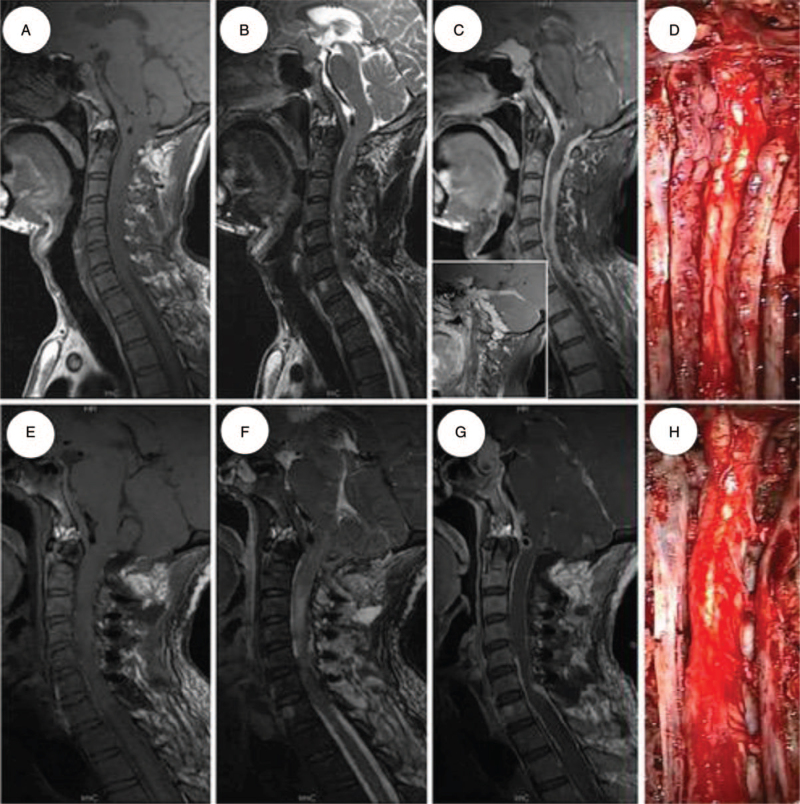
Preoperative (A-C) magnetic resonance image (MRI) shows the lesion extends from tentorium cerebella to the sixth cervical spinal canal and enveloping the cervical spinal cord with isointense on T1-weighted image (T1WI) and T2-weighted image (T2WI)(A-B), and enhanced remarkably and homogeneously after infusion of Gd-DTPA (C), and postoperative (E-G) MRI revealed the tumor in the cervical spinal canal was almost resected, without excision of the lesion crawl on the tentorium cerebella and basis crania. Intraoperative photo (D, H): The tumor grew along the meninges displayed an irregular shape but clear boundaries with the spinal cord. The color of the tumor was greyish white with more than usually vascular, the cervical cord was compressed (D). After excision of the mass in the cervical canal, the spinal cord was decompressed, and cervical spinal cord roots are clear (H). Gd-DTPA = gadolinium-diethylene triamine pentaacetic acid.

Magnetic resonance imaging of the cervical spinal cord showed a diffuse extramedullary mass creeping on the tentorium and skull base meninges along the clivus down to the sixth cervical spinal meninges. The cervical spinal cord was enveloped and pressed. The lesion was isointense on T1-weighted imaging and T2-weighted imaging and enhanced remarkably and homogeneously after infusion of gadolinium-diethylene triamine pentaacetic acid (Fig. 1 A-C). Laboratory findings showed total serum protein was 62.3 g/L (normal 65–85 g/L), and serum albumin was 36.3 g/L (normal 40–55 g/L). Serum globulins included an IgE of 990.38 IU/mL (normal 0.1–150 IU/mL) and IgM of 2880 mg/L (normal 700–2200 mg/L), with normal quality of IgG. Routine blood examination showed mild anemia, with a red blood cell count of 3.64 × 10^12^/L and hemoglobin level of 108 g/L. Urinary sediment quantitative analysis showed red blood cell 13286/μL (normal 0–1L/uμL) and white blood cell 952/μL. Computed tomography of the patient's chest, abdomen, and pelvis showed no abnormalities. Based on the patient's cervical spinal cord MRI and blood test results, we preliminarily inferred that the patient's lesion was a meningioma. Because the patient's lesions were extensive and located at the base of the skull, the prognosis of this patient was estimated to be poor.

### 2.3 Operation and pathological findings

**Figure 2:**
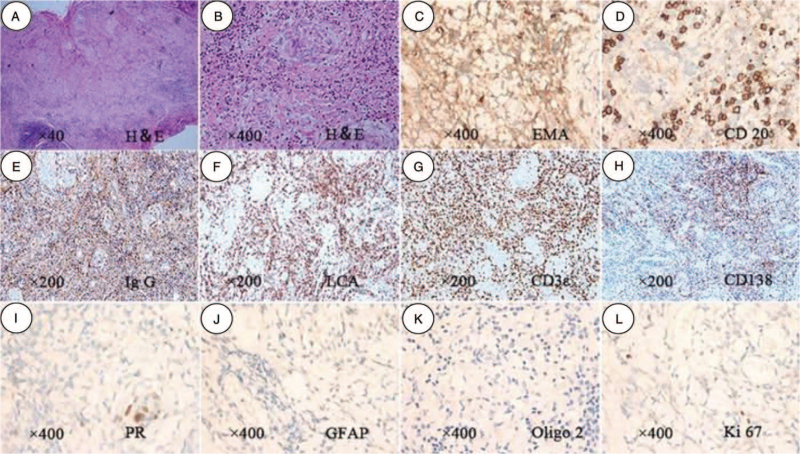
Photomicrographs demonstrating the histological features of the mass. Hematoxylin and eosin (H&E) (A-B) staining showed granulation-like tissue composed of inflammatory cells and vascular proliferation. The tumor cells were positive for EMA (C), CD20 (D), IgG (E), LCA (F), CD3ε (G), CD138 (H), and PR (I), negative for GFAP (J) and oligo2 (K). The Ki-67 (L) proliferative index of the tumor was less than 1%. MA = epithelial membrane antigen, GFAP = Glial fibrillary acidic protein.

We considered that the patient had a urinary tract infection, and antibiotics were administered. When the urinary tract infection was cured, we partially removed the tumor through suboccipital craniotomy, C1 laminectomy, and C2-C6 laminoplasty. During the operation, a greyish-white mass was seen attached to the dura mater extending from the clivus to the sixth cervical spinal cord, with more blood vessels than usual (Fig. 1D). Following subtotal tumor resection (Fig. 1H) and primary dural closure, the lamina was replaced and attached to the titanium microplates. Postoperative MRI revealed that the tumor in the cervical spinal canal was almost resected, without excision of the lesion creeping on the tentorium cerebella and base crania (Fig. 1E-G). Histopathological examination of the mass showed meningothelial areas admixed with lymphocytes and plasma cells. The H&E staining from specimens showed a large number of nuclear accumulations (Fig. 2 A, B). Immunohistochemically, the tumor cells were positive for epithelial membrane antigen, progesterone receptor, IgG CD138, and CD3ε (Fig. 2C, I, E, H, G). A large number of lymphocytes were positive for LCA and CD20 (Fig. 2F, D). Glial fibrillary acidic protein, cytokeratin, and oligo2 were negative (Fig. 2J, K). The Ki-67 proliferative index of the tumor was less than 1% (Fig. 2L). The magnetic resonance imaging report and immunohistochemical results indicated that the lesion was a lymphoplasmacyte-rich meningioma. According to the WHO, the mass was categorized as a grade I tumor in the classification of tumors of the CNS.^[3]^

### 2.4 Postoperative course

Postoperatively, the patient developed dyspnea and pneumonia. After 2 weeks of active treatment, the patient died of worsening pneumonia.

## 3 Discussion

LPRM, a rare subtype of meningioma, was first reported by Banerjee in 1971. According to the WHO classification of CNS tumors, LPRM is characterized by predominant infiltration of lymphocytes and plasma cells with scattered small entrapped meningothelial whorls.^[3]^ The pathogenesis of LPRM is not clearly understood, and controversy still exists regarding the infiltration of LPRM lymplasmacytes. Monocellular infiltrates in brain tumors have been widely reported in various types of tumors. There are only 2 subtypes of meningioma, LPRM and choroid meningioma, with obvious type B lymphocyte infiltration.^[5]^ Whether the inflammatory lesions are indeed primary neoplastic in nature or merely granulomatous with a secondary meningeal reaction is under debate. Weidenheimet et al believed that lymphoplasmacytic infiltrations are an immunological stimulation of tumor antigenicity.^[6]^ While Gregorios et al considered that the inflammatory cells could derive from differentiation of a totipotent cluster of mesenchymal cells, and it will produce chronic and continuous inflammation once verified the neoplastic nature of the meningothelial component.^[7]^

The sites of the involvement of LPRM seem multiple, and most cases perform as single nodularity mass with atypical clinical appearance.^[8]^ We reviewed the relevant literature and consulted about 60 previously published reports of lymphoplasmacyte-rich meningiomas. Our case presented the widest range lesion, which involves saddle area, tentorium cerebella, and along the meninges of the skull base from clivus down to the sixth cervical spinal cord.

Due to its atypical characteristics, some CNS neoplasms or masses, such as choroid meningioma, plasma cell granuloma, solitary plasmacytoma, and others can mimic an LPRM; thus, the preoperative diagnosis of LPRM is often difficult.[^9^,^10^,^11^] Liu et al^[12]^ believed the blood-brain barrier could be damaged and those MRI findings could be used to distinguish between LPRM and other common meningiomas. In addition, occasional cases may show blood abnormalities, such as hyperglobulinemia and/or anemia, which could be used for further distinguish diagnosis. It is generally confessed that blood abnormalities are dependent on plasmacyte infiltrates because abnormalities disappear after complete resection of the tumor and reappear with recurrence.[^12^,^13^,^14^,^15^] However, accurate diagnosis often depends on the postoperative pathologic analysis. The special histological characteristics of LPRM are abundant tumor cells and plasma cells that form a cuff-like structure around the vascular tissue.

Surgical excision is the primary treatment for LPRM. Most of these cases show favorable clinical outcomes with no recurrence or metastasis.[^13^,^14^,^16^,^17^] Postoperative adjuvant treatments are not usually performed if there are no recurrent or residual tumors. There are only 2 cases in the literature in which patients accept adjuvant treatment.[^14^,^18^] In a study by Horten in 1979,^[14]^ the patient accepted radiation (3000 rad) and chemotherapy (methotrexate); however, the patient gradually deteriorated to spastic quadriparesis and died two years after the onset of illness. Another case was reported by Wang in 2013, in which Gamma-Knife radiosurgery was successfully used to treat residual tumor.^[18]^

Based on the wide range of tumor growth in this patient, we believe that the tumor could not be completely resected. Radical tumor resection, which induces severe neurological deficits, is unnecessary.^[19]^ Although lymphoid plasma cell meningioma is a benign tumor, in addition to active surgical treatment, the residual part of the tumor after surgery can be treated with radiotherapy or Gamma-Knife radiosurgery .^[18]^ At the same time, according to the scoring system based on the quantification of 2 lymphocyte populations (CD3 and CD8), both at the tumor center and the invasive margin in the pathological specimen of the patient, this type of tumor can be defined as a hot tumor.^[20]^ Therefore, immunotherapy may be effective for this type of tumor. Chen et al^[21]^ reported immune checkpoint blockers and chimeric antigen receptor T-cells have led to a paradigm shift in current oncology practices and provided new treatment options for patients. Indeed, many preclinical studies have recently demonstrated that administering immunotherapy before definitive surgical resection of tumors can produce significant clinical benefits.[^22^,^23^,^24^] Therefore, for patients with lymphoplasmacyte-rich meningioma, immunotherapy before surgery may be of great help in minimizing the scope of surgical resection and shortening the operation time.^[21]^

## 4 Conclusion

Lymphoplasmacyte-rich meningiomas with extensive masses are extremely rare. Therefore, it is difficult to make a definitive preoperative diagnosis. Although previous studies have shown that complete surgical resection can reduce the recurrence rate, formulating an individualized surgical plan is necessary. In this case, we performed a subtotal resection of the tumor by suboccipital craniotomy, C1 laminectomy, and C2-C6 laminoplasty. Most cases had a favorable outcome; however, this patient developed dyspnea and pneumonia after the operation. After 2 weeks of active treatment, the patient died of worsening pneumonia. We suspect that the patient's dyspnea was caused by edema of the cervical spinal cord (Fig. 1F) after surgical decompression. Based on the characteristics of LPRM, immunotherapy may be a new treatment option in the future. If immunotherapy can achieve a good outcome, surgical resection of the tumor will no longer be the first choice for these patients with LPRM.

## Author contributions

**Conceptualization:** Yuelong Wang, Haifeng Chen, Siqing Huang, Jianguo Xu.

**Data curation:** Bin He, Yuelong Wang.

**Writing – original draft:** Han Wang.

**Writing – review & editing:** Haifeng Chen, Siqing Huang, Jianguo Xu.
